# Dimethylthiourea protects against chlorine induced changes in airway function in a murine model of irritant induced asthma

**DOI:** 10.1186/1465-9921-11-138

**Published:** 2010-10-06

**Authors:** Toby K McGovern, William S Powell, Brian J Day, Carl W White, Karuthapillai Govindaraju, Harry Karmouty-Quintana, Normand Lavoie, Ju Jing Tan, James G Martin

**Affiliations:** 1 Meakins Christie Laboratories, Department of Medicine, McGill University, Montreal, Quebec, Canada; 2 Department of Pediatrics, National Jewish Health, Denver, Colorado, USA

## Abstract

**Background:**

Exposure to chlorine (Cl_2_) causes airway injury, characterized by oxidative damage, an influx of inflammatory cells and airway hyperresponsiveness. We hypothesized that Cl_2_-induced airway injury may be attenuated by antioxidant treatment, even after the initial injury.

**Methods:**

Balb/C mice were exposed to Cl_2 _gas (100 ppm) for 5 mins, an exposure that was established to alter airway function with minimal histological disruption of the epithelium. Twenty-four hours after exposure to Cl_2_, airway responsiveness to aerosolized methacholine (MCh) was measured. Bronchoalveolar lavage (BAL) was performed to determine inflammatory cell profiles, total protein, and glutathione levels. Dimethylthiourea (DMTU;100 mg/kg) was administered one hour before or one hour following Cl_2 _exposure.

**Results:**

Mice exposed to Cl_2 _had airway hyperresponsiveness to MCh compared to control animals pre-treated and post-treated with DMTU. Total cell counts in BAL fluid were elevated by Cl_2 _exposure and were not affected by DMTU treatment. However, DMTU-treated mice had lower protein levels in the BAL than the Cl_2_-only treated animals. 4-Hydroxynonenal analysis showed that DMTU given pre- or post-Cl_2 _prevented lipid peroxidation in the lung. Following Cl_2 _exposure glutathione (GSH) was elevated immediately following exposure both in BAL cells and in fluid and this change was prevented by DMTU. GSSG was depleted in Cl_2 _exposed mice at later time points. However, the GSH/GSSG ratio remained high in chlorine exposed mice, an effect attenuated by DMTU.

**Conclusion:**

Our data show that the anti-oxidant DMTU is effective in attenuating Cl_2 _induced increase in airway responsiveness, inflammation and biomarkers of oxidative stress.

## Introduction

Respiratory health is adversely affected by exposure to strong irritant substances such as chlorine (Cl_2_) or ozone [1]. A single, acute exposure of persons to Cl_2 _in an industrial or domestic context may trigger asthma in a proportion of those exposed and is termed irritant-induced asthma [2,3]. High dose exposures may lead to acute lung injury and death [4]. Although the mechanism of the induction of asthma by irritants is uncertain, this form of asthma may be a significant contributor to the current rising prevalence of this disease. Some of the irritants that induce symptoms of asthma such as ozone and Cl_2 _cause oxidant injury, in particular to the airway epithelium. Desquamation of the airway epithelium and prolonged sub-epithelial inflammation accompanied by airway hyperresponsiveness has been documented following a single acute Cl_2 _inhalational exposure [5]. Epithelial shedding may adversely affect barrier function of the epithelium and may diminish the influence of epithelial-derived bronchodilator substances such as nitric oxide [6]. Cl_2 _is a highly reactive substance and has been documented to cause airway injury in mice that is associated with oxidant stress, as evidenced by the finding of peroxynitrite in the airway tissues and carbonylation of proteins [7]. There may be additional contributions to oxidant injury through activation of inflammatory cells [8]. The causative role of oxidative stress in the changes in airway function and airway inflammation caused by a potent oxidant like Cl_2 _is relatively under-investigated. Recently a combination of anti-oxidants (ascorbic acid, desferroxamine and N-acetylcysteine) was found to attenuate signs of respiratory dysfunction, in particular gas exchange and microvascular leak, in the rat [9].

The current study was designed to examine the relationship between oxidant damage, airway hyperresponsiveness and inflammation caused by Cl_2 _by testing the efficacy of an anti-oxidant in protecting against these effects. For this purpose we used dimethylthiourea (DMTU), an oxygen metabolite scavenger [10], that is highly cell-permeable [11-13]. We also wished to examine the effects of Cl_2 _on markers of oxidative stress and whether DMTU attenuated these effects. We hypothesized that treatment with DMTU would ameliorate the inflammatory and pathophysiological effects induced by Cl_2 _gas exposure whether administered before or after exposure.

## Methods

### Animals and protocol

Male Balb/C mice (18-22 g) were purchased from Charles River (Wilmington, Massachusetts) and housed in a conventional animal facility at McGill University. Animals were treated according to guidelines of the Canadian Council for Animal Care and protocols were approved by the Animal Care Committee of McGill University.

Mice were exposed to either room air (control) or Cl_2 _gas diluted in room air for 5 minutes using a nose-only exposure chamber. An initial experiment was performed to assess an exposure level required to effect changes in airway responsiveness to methacholine (MCh) that was well tolerated by the animals. For this purpose we exposed mice to 100, 200 or 400 ppm Cl_2_, and 24 hours later we performed MCh challenge and removed the lungs for histological analysis. Based on the results of this experiment we tested the effects of DMTU on animals exposed to 100 ppm Cl_2_. The control mice were exposed to room air (Control; n = 6) and test mice were exposed to Cl_2 _(Cl_2_; 100 ppm; n = 6) with DMTU (100 mg/kg) treatment intraperitoneally either one hour before (DMTU/Cl_2_; n = 6) or one hour after Cl_2 _exposure (Cl_2_/DMTU; n = 6). DMTU was prepared fresh prior to each exposure and a dose of 100 mg/kg in 500 μL of sterile phosphate buffered saline (PBS) was administered i.p. either one hour before or one hour following exposure to Cl_2_. Control (air exposed) mice received 500 μL PBS i.p and Cl_2 _exposed mice received 500 μL PBS i.p. either one hour before or one hour following exposure. We chose the dose of DMTU based on previous observations of efficacy against an oxidant pollutant in mice [11]. At 24 hours after Cl_2 _exposure, lung function measurements including responsiveness to aerosolized MCh were performed and bronchoalveolar lavage fluid was obtained for assessment of inflammatory cell counts, total protein, nitrate/nitrite (nitric oxide) and glutathione levels. The lungs were removed for analysis of carbonylated proteins and 4-hydroxynonenal (4-HNE). Measurements of inflammatory cell counts and glutathione levels in BAL fluid were made also at 10 min and at 1 hr after Cl_2_. Following exposure animals were returned to the animal facility and allowed food and water *ad libitum*.

### Exposure to Cl_2_

Mice were restrained and exposed to Cl_2 _gas for 5 minutes using a nose-only exposure device. Cl_2 _gas was mixed with room air using a standardized calibrator (VICI Metronics, Dynacalibrator^®^, model 230-28A). The Cl_2 _delivery system has two main components, a gas generator, which includes a heated permeation chamber and air flow generator. Dynacal permeation tubes designed specifically for operation with the Dynacalibrator were used and contain the Cl_2_. The permeation chamber and air flow generator control accuracy of the Cl_2 _generated to within 1-3% of the desired concentration (manufacturer's specifications). Within the gas chamber, permeation tubes containing Cl_2 _are housed for gas delivery. The Teflon permeation tubes contain Cl_2 _in both gas and liquid phases. When the tube is heated the Cl_2 _reaches a constant and increased vapor pressure and it permeates the tube at a constant rate. The desired concentration is delivered at an appropriate flow rate, as specified by the manufacturer. The device is attached to the exposure chamber and allowed to calibrate for 30 minutes until the optimum temperature of 30°C is reached and the Cl_2 _flow is constant. Following removal of the animals from the exposure chamber, the chamber was continually flushed with the gas mix to ensure that the desired concentration of Cl_2 _was maintained.

### Evaluation of Respiratory Responsiveness

Mice were sedated with an intraperitoneal (i.p) injection of xylazine hydrochloride (8 mg/kg) and anaesthetized with i.p. injection of pentobarbital (30 mg/kg). Subsequently, the animal was tracheostomized using at 18 gauge cannula and connected to a small animal ventilator (FlexiVent, Scireq, Montreal, Canada). Muscle paralysis was induced with pancuronium bromide (0.2 mg/kg i.p.). The mice were ventilated in a quasi-sinusoidal fashion with the following settings: a tidal volume of 10 mL/kg, maximum inflation pressure of 30 cmH_2_0, a positive end expiratory pressure (PEEP) of 3 cmH_2_0 and a frequency of 150/min. Following an equilibration period of 3 minutes of tidal ventilation two lung inflations to a transrespiratory pressure of 25 cm H_2_O were performed and baseline measurements were taken. The respiratory mechanics were estimated using a single compartment model and commercial software (Scireq). Baseline was established as the average of three perturbations. Following establishment of baseline, MCh was administered using an in-line nebulizer (Aeroneb Lab, standard mist model, Aerogen Ltd, Ireland) and progressively doubling concentrations ranging from 6.25 to 50 mg/ml were administered over 10 seconds synchronously with inspiration. Six perturbations were calculated at each dose of MCh to establish the peak response. The highest value was kept for analysis subject to a coefficient of determination above 0.85. Respiratory system resistance (Rrs) and respiratory system elastance (Edyn) were determined before challenge and after each dose of MCh.

### Bronchoalveolar Lavage Fluid Analysis

Following euthanasia (60 mg/kg pentabarbital, i.p.), the lungs were lavaged with 600 μl of sterile saline, followed by four separate aliquots of 1 ml each as previously described [7]. The first 600 μlmL aliquot of BAL fluid was centrifuged at 1500 rpm for 5 minutes at 4°C and the supernatant was retained for measurements of nitric oxide, glutathione levels and protein levels using a Bradford Assay. The separate 1 mL aliquots were spun at 1500 rpm for 5 min at 4°C and the supernatant removed. The cell pellets were pooled for differential cell counts using 100 μl of the re-suspended cells. Cytospins were prepared, air-dried and stained (Diff-Quik^® ^method, Medical Diagnostics, Düdingen, Germany). A differential cell count was determined on a minimum of 300 cells.

### Histology

Following harvesting, the lungs were perfused with saline until the effluent was clear. The right lung was inflated with 1 mL 10% buffered formalin, fixed overnight with formalin. Tissues were embedded in paraffin blocks, cut into 5 μm sections and stained with hematoxylin and eosin. Sections were evaluated for epithelial morphological changes. The absolute number of epithelial cells in the airways was determined by counting cells on hematoxylin and eosin stained slides at 200× magnification and data were expressed as the number of epithelial cells per mm of basement membrane perimeter (P_BM_). Epithelial cell height was determined by measuring the distance between the basement membrane and the top of the epithelial cell in the four quadrants for each airway and averaged.

### Measurement of Nitrite/Nitrate in BAL

For the evaluation of nitric oxide, 0.6 N trichloroacetic acid was added to the supernatant of the BAL fluid to give a final concentration of 0.12 N to precipitate any protein. Samples were centrifuged for 10 minutes at 10,000 RPM followed by removal of the supernatant for analysis using previously described methods [7]. Total NO_x _was measured in BAL as an index of NO production using the Griess reaction. Briefly, 80 μl of sample were pre-incubated with 20 μl of NO_3 _reductase and 10 μl of its enzyme cofactor for 3 h at room temperature and then incubated with 100 μl of Griess reagent for 10 min. NO_x _concentrations were determined using standard curves obtained from different concentrations of NaNO_2 _or NaNO_3_. Absorbance was measured at 540 nm with a plate reader (SLT 400 ATC; SLT Lab Instruments, Salzburg, Austria). No NO_x _was detected in saline solutions using this assay.

### Carbonylated protein residues (Oxyblot)

An Oxyblot was performed on left lung tissue extracts taken 24 hours following Cl_2 _challenge. Extracted proteins were denatured with 12% sodium dodecylsulfate (SDS) before derivatization with the addition of DNPH (2,4-dinitrophenylhydrazone-hydrazone). DNPH-derivatised proteins were separated on a 10% SDS-PAGE gel at 140 V for 2 h. Proteins were then electrophoretically transferred onto polyvinylidene difluoride (PVDF) membrane with 11.6 mM Tris (Fisher), 95.9 mM glycine (Fisher) and 20% methanol (Fisher) at 25 V for 2 h. Membranes were then blocked with 1% bovine serum albumin-TTBS solution (0.02 M Tris base, 0.5 M NaCl, and 0.1% of Tween 20; Sigma) and were probed for 90 min with rabbit anti-DNP antibody (Intergen Company, Purchase, NY). The membranes were then rinsed in TTBS and incubated with HRP-conjugated goat anti-rabbit IgG (Intergen Company, Purchase, NY) for 1 h. A chemiluminescence detection system (ECL Plus; Amersham), Hyperfilm (Amersham), and Fluorochem 8000 software (Alpha Innotech Corporation, San Leandro, CA) were used for antibody detection and quantification by densitometry.

### Lung 4-hydroxynonenal (4-HNE) assay

All reagents were from Sigma-Aldrich (St. Louis, MO, USA) unless otherwise stated. Frozen tissue, or a known amount of 4- HNE standard (Cayman Chemical, Ann Arbor, MI, USA), was placed in 2 ml of cold methanol (Thermo Fisher) containing 50 μg/ml butylated hydroxytoluene, with 10 ng d3-4-HNE (Cayman Chemical) internal standard added just before homogenization with the Ultra-Turrax T25 (Thermo Fisher). An EDTA solution (1 ml of 0.2 M, pH 7) was added. Derivatization was accomplished by the addition of 0.2 ml of 0.1 M HEPES containing 50 mM O-(2,3,4,5,6-pentafluorobenzyl)hydroxylamine hydrochloride, pH 6.5. The mixture was then vortexed and held at room temperature. After 5 min, 1 ml of hexanes (Thermo Fisher) was added, and the samples were shaken vigorously. Brief centrifugation was performed to achieve phase separation and the O-pentafluorobenzyl-oxime derivatives were extracted from the upper hexane layer. The sample was dried under a stream of N2 gas and further derivatized into trimethylsilyl ethers by the addition of 15 μl each of pyridine and N, O bis(trimethylsilyl)trifluoroacetamide. The samples were vortexed and heated to 80°C for 5 min and then analyzed for 4-HNE content by GC/MS. GC/MS analysis was performed using a Focus GC coupled to a DSQ II mass spectrometer and an AS 3000 autosampler (Thermo Fisher).A15-m TR-5MS column (0.25-mm i.d., 0.25-μm film thickness; Thermo Fisher) was used with ultrahigh-purity helium as the carrier gas at a constant flow rate of 1.0 ml/min. Two microliters of sample was injected into the 270°C inlet using split mode with an injection ratio of 10 and a split flow of 10 ml/min. The initial oven temperature was 100°C and then ramped to 200°C at 15°C/min, followed by an increase in temperature to 300°C at 30°C/min, and held for 1 min. The MS transfer line temperature was held constant at 250°C and the quadrupole at 180°C. Analysis was done by negative-ion chemical ionization using 2.5 ml/min methane reagent gas. Ions were detected using SIM mode with a dwell time of 15.0 ms for each fragment of 4-HNE at m/z 152, 283, and 303, and d3-4-HNE at m/z 153, 286, and 306. Under these conditions, the larger, second peak of the two 4-HNE isomers was used for quantification and exhibited a retention time of 7.18 min, which was just preceded by the elution of d3-4-HNE at 7.17 min. Quantification was performed using a standard curve generated by graphing the area ratio of 4-HNE to d3-4-HNE versus concentration.

### Measurement of glutathione (GSH and GSSG) in BAL fluid and cells

BAL fluid from control, chlorine exposed and DMTU pre-treated chlorine exposed mice was collected for glutathione evaluation by HPLC. Both glutathione (GSH) and glutathione disulfide (GSSG) were measured to determine if GSH had converted to GSSG. As GSH is found almost exclusively in its reduced form, a conversion to GSSG, which his inducible following oxidative stress, would indicate an increase in oxidative stress in the lung. BAL samples were collected at 10 minutes, one hour and 24 hours after Cl_2 _challenge. Phosphoric acid (60 μL; 1 M) was added to BALF samples to prevent GSH degradation. BAL was centrifuged at 1500 RPM for 5 minutes, and the supernatant was removed for evaluation of extracellular GSH/GSSG and 150 μL of PBS and 15 μL 1 M phosphoric acid added was used to reconstitute the pellet for analysis of intracellular GSH and GSSG. CHAPPS (150 μL; 6 mM) was added to lyse the cells. GSH and GSSG were measured by RP-HPLC using a post-column derivatization procedure modified from the literature [14]. GSH and GSSG levels were determined in 50 μl aliquots by RP-HPLC using a gradient prepared from 0.05% trifluoroacetic acid (TFA) in water (solvent C) and 0.05% TFA in acetonitrile (solvent D) as follows: 0 min, 0% D; 10 min, 15% D. The flow rate was 1 ml/min and the stationery phase was a column (150 × 4.6 mm) of Ultracarb ODS (31% carbon loading; 5 μm particle size; 150 × 4.6 mm; Phenomenex, Torrance, CA). The eluate from the column was mixed with o-phthalaldehyde (370 μM) in 0.2 M tribasic sodium phosphate, pH 12, which was pumped into a T-fitting using an auxiliary pump (Waters Reagent Manager). The mixture then passed through a loop of PEEK tubing (6 m × 0.5 mm, i.d.; volume, 1.2 ml) that was placed in a water bath at 70°C. Under these conditions both GSH and GSSG are converted to a fluorescent isoindole adduct, which is measured using excitation and emission wavelengths of 336 and 420 nm, respectively. Prior to introduction into the fluorescence detector (Waters model 2475 Multi wavelength Fluorescence Detector), the mixture was cooled in a small ice-water bath and passed through a filter containing an OptiSolv 0.2 μm frit (Optimize Technologies). The amounts of GSH and GSSG were determined from a standard curve using the authentic compounds as external standards.

### Statistical analysis

Data were analyzed using an analysis of variance and for *post hoc *comparisons of means a Newman-Keuls test was used. A p < 0.05 was accepted as significant. All values are expressed as the mean + one standard error of the mean.

## Results

### Concentration-dependent changes in airway responsiveness following Cl_2_

**Figure 1:**
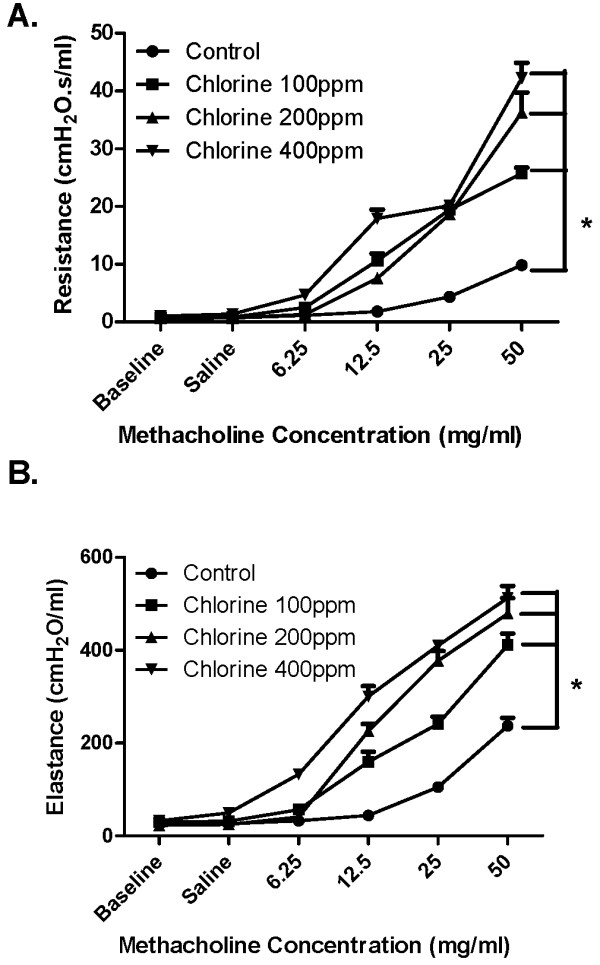
**Dose-response effect of Cl_2 _on respiratory responsiveness to methacholine**. Mice were either unchallenged (Control; n = 6) or challenged with 100 (n = 6), 200 (n = 6) or 400 (n = 6) ppm Cl_2 _gas. After 24 h, total respiratory system resistance (A) and respiratory system elastance (B) in response to saline (Sal) and doubling doses of MCh were assessed using a small animal ventilator (FlexiVent). Baseline (Base) values obtained from untreated mice are shown for comparison. Mice treated with all three concentrations of Cl_2 _showed significantly higher respiratory system resistance and at 12.5, 25, and 50 mg/ml of MCh as compared with control. * p < 0.05, n = 6 per group.

To establish a suitable submaximal concentration of Cl_2 _for subsequent experiments animals were exposed to 100 ppm, 200 ppm or 400 ppm of Cl_2 _for 5 minutes. The next day, the animals were challenged with doubling doses of MCh ranging from 6.25 to 50 mg/ml. Respiratory system resistance (Figure 1A) and elastance (Figure 1B) were evaluated. There was a dose-dependent increase in responsiveness to MCh reflected in both of the above parameters of lung function.

### Histological changes in the airways after Cl_2 _exposure

**Figure 2:**
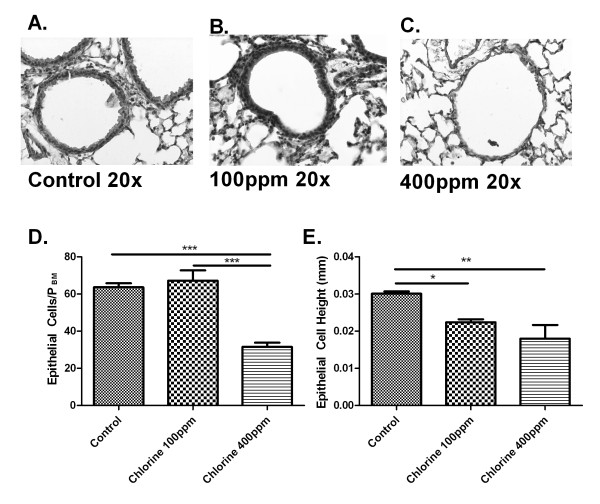
**Effects of Cl_2 _on airway histology**. Twenty-four hours following Cl_2 _exposure lungs were collected, paraffin embedded and lung sections cut (5 μM). Sections were then stained with hematoxylin and eosin. Representative pictures of airway sections from control mice (A) mice treated with 100 (B), or 400 ppm (C) Cl_2_. Total epithelial cells were quantified in each airway and corrected for P_BM _and showed no difference between control and 100 ppm, but significantly fewer epithelial cells at 400 ppm (D). Epithelial cell height was also calculated and showed that mice given 100 ppm and 400 ppm had shorter epithelial cells than control (E).

The effects of Cl_2 _on airway architecture were assessed on hematoxylin and eosin stained lung sections obtained 24 hours after exposure (Figure 2). Lower concentrations of Cl_2 _(100 ppm and 200 ppm) did not result in any detectable change under light microscopy to the airway epithelium (Figure 2A and 2B). There was an obvious thinning of the airway epithelium at a concentration of 400 ppm (Figure 2C). There were statistically significant differences observed in epithelial cell height caused by exposure to Cl_2 _(Fig 2E). We also quantified the number of epithelial cells in the airway walls. While there was no significant difference in cell following exposure to Cl_2 _at 100 ppm compared to control (Figure 2D), at 400 ppm, there were fewer epithelial cells compared to both control and 100 ppm (Fig 2D). Given the lack of gross histological change induced by 100 ppm of Cl_2 _we chose to perform further studies using this concentration.

### Effect of DMTU on MCh responsiveness following Cl_2 _challenge

**Figure 3:**
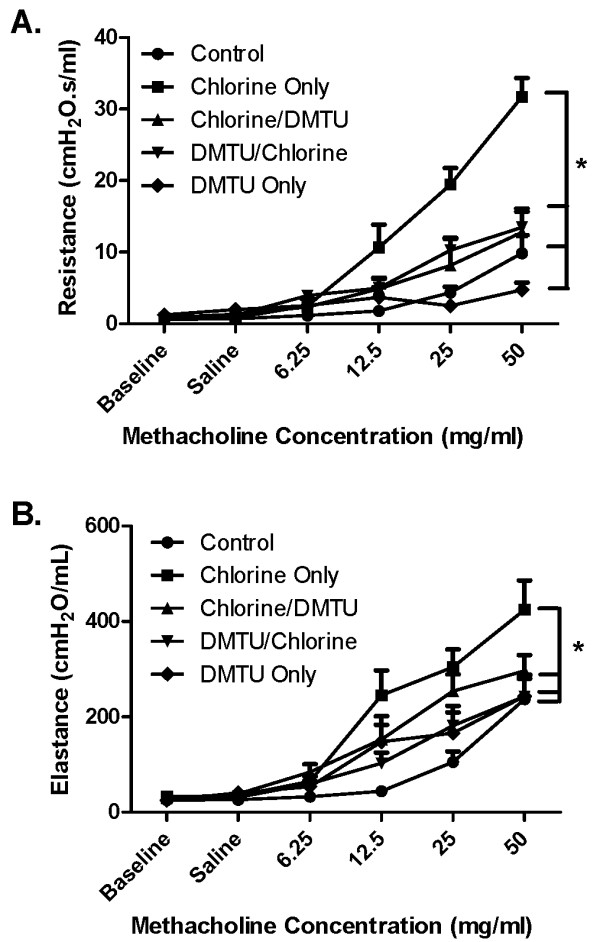
**Effects of Cl_2 _on methacholine respiratory system resistance and elastance**. Panel A shows the effects of Cl_2 _exposure on total respiratory system resistance in mice that were treated with before and 1 hour after exposure with DMTU. A two-way ANOVA showed that there is a significant difference between mice pre- or post-treated with DMTU when compared to animals receiving Cl_2 _only. Panel B shows the effects of Cl_2 _exposure and DMTU treatment on total respiratory system elastance. DMTU/Cl_2 _treated animals had elastance levels similar to control whereas Cl_2 _only treated mice had significantly higher values compared to control: n = 6 per group; * p < 0.05.

Airway responses to increasing doses of MCh (6.25-50 mg/ml) were elevated 24 h following Cl_2 _challenge (Figure 3A). This effect was attenuated by administration of DMTU given both prior to and post Cl_2_-exposure. Changes in respiratory system elastance in response to MCh paralleled those observed for resistance (Figure 3B). DMTU alone had no significant effect on MCh responsiveness.

### Changes in bronchoalveolar lavage cells after Cl_2 _gas exposure

**Figure 4:**
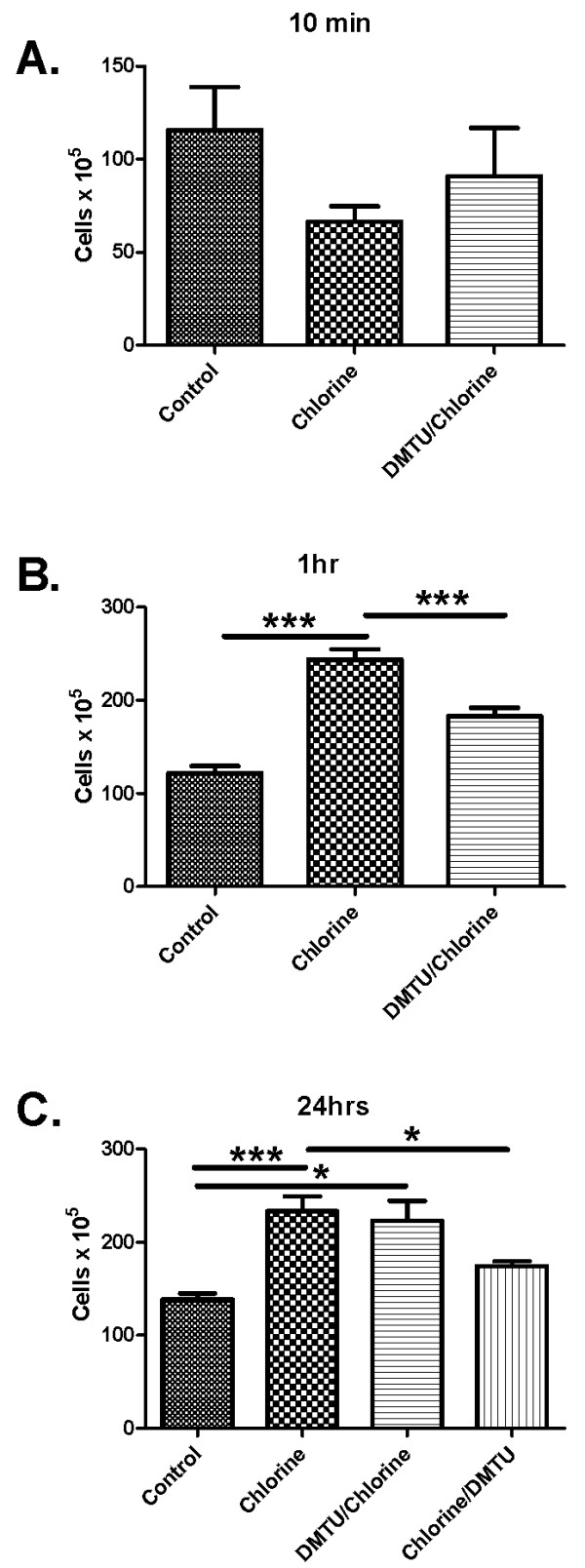
**Effects of Cl_2 _exposure on the numbers of cells in BAL fluid**. Data for control and Cl_2 _exposed animals that were sacrificed 10 minutes (A), 1 hour (B) and 24 hours (C) after Cl_2 _exposure. Cl_2 _exposure caused a significant increase in total leukocytes compared to controls at 1 hour and 24 hours, the effect of which was attenuated by pre-treatment with DMTU at one hour and post treatment with DMTU at 24 hours. (n = 6 per group; * p < 0.05., **p < 0.01, ***p < 0.001).

**Figure 5:**
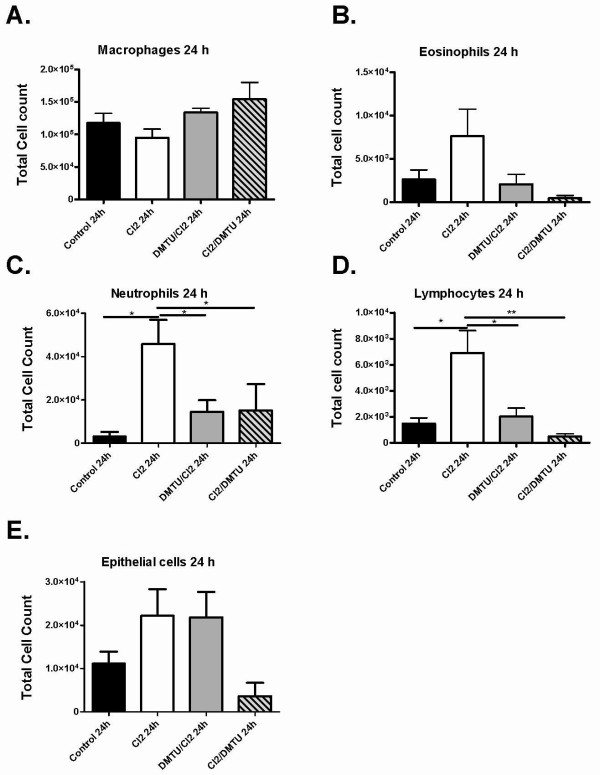
**Cellular composition of BAL fluid following Cl_2 _exposure**. Differential cell counts were done at 10 minutes and 24 hours. No cell subset was significantly different at 10 min (data not shown). At 24 hours neutrophils and lymphocytes were significantly elevated in Cl_2 _groups. Treatment with DMTU was limited increases in these cell types. There was no difference between control and DMTU treated groups. Control (n = 9), Cl_2 _100 ppm (n = 7), DMTU/Cl_2 _(n = 7), Cl_2 _/DMTU (n = 6); * <0.05.

To assess the effects of Cl_2 _on airway inflammation and epithelial cell shedding bronchoalveolar lavage was performed at 10 minutes, one hour and at 24 hours after Cl_2 _exposure. The fluid recovered by BAL averaged 75% of the volume instilled and did not differ significantly among the groups. Total cell counts were not significantly different at 10 minutes after exposure to Cl_2 _(Figure 4A) but were significantly increased in Cl_2 _treated groups by one and 24 hours compared to control (Figure 4B and 4C). At one hour, pre-treatment with DMTU reduced the total number of inflammatory cells present in the airways compared to Cl_2 _only mice. At 24 hours, total cell counts were persistently elevated after Cl_2 _and were attenuated only in mice post-treated with DMTU after Cl_2 _exposure (Figure 4C). Cl_2 _caused a significant increase in neutrophils and lymphocytes 24 hours following challenge, an effect attenuated by both pre- and post-treatment with DMTU (Figure 5C and 5D). There were no significant changes in any of the cell subsets at 10 mins (Figure 5A, B and 5E).

### Changes in protein level following Cl_2 _exposure

**Figure 6:**
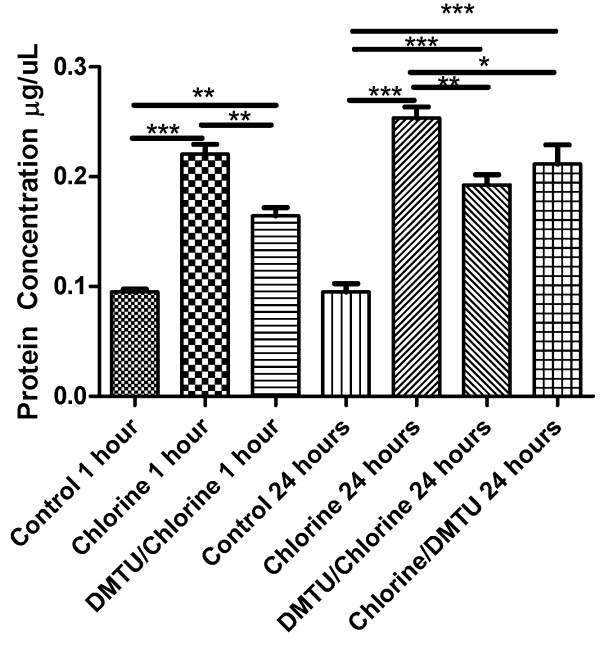
**Effects of Cl_2 _exposure and DMTU treatment on BAL fluid protein**. Protein levels in BAL fluid were assessed by Bradford assay. There was a significant increase in total protein at 1 and 24 hours after Cl_2 _exposure. Pre-treatment with DMTU attenuated the increase in protein at both time points and at 24 hours when given one hour post- Cl_2 _exposure. (n = 6-9/group; * p < 0.05, **p < 0.01, ***p < 0.001).

We measured the total protein level in BAL fluid harvested at 1 and 24 hours after Cl_2 _exposure to assess the effects of Cl_2 _on cell damage and protein levels. At both time points following Cl_2 _exposure there was a significant increase in total protein in the BAL fluid as assessed by the Bradford assay. Treatment with DMTU, both before and after Cl_2 _exposure reduced protein levels in BAL (Figure 6).

### Effects of Cl_2 _on markers of oxidative stress

**Figure 7:**
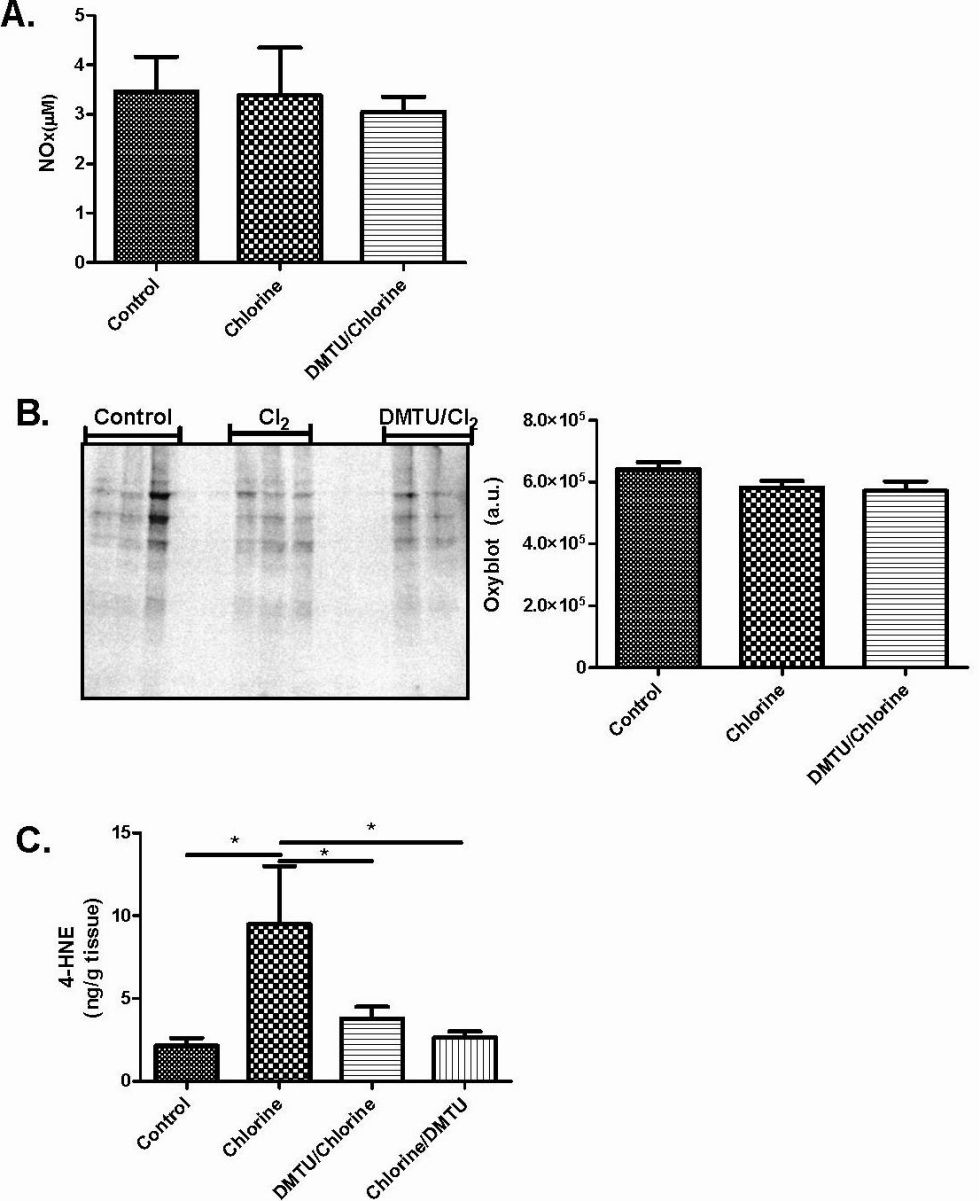
**Effects of Cl_2 _exposure and DMTU treatment on markers of oxidative stress**. (A) Nitric oxide was also measured 24 hours following Cl_2 _exposure using a Griess reaction and no significant change was seen between any of groups. (B) Twenty-four hours following Cl_2 _exposure BAL was collected and an OxyBlot was performed on lung tissue homogenates to detect carbonylated proteins. No significant differences were detected among the groups. (C) Twenty-four hours following chlorine exposure, lungs were collected for 4-HNE analysis. Chlorine caused a significant increase in 4-HNE levels over control and DMTU treated groups. There were no differences between DMTU groups and baseline. (n = 6-10, * p < 0.05).

Nitric oxide concentrations were determined using the Griess reaction and no significant change was seen between any of groups 24 hours following Cl_2 _challenge (Figure 7A). An OxyBlot was performed on lung extracts to detect proteins modified by oxygen metabolites 24 hours following Cl_2 _exposure. Levels of carbonylation were quantified by densitometry and no substantial difference was seen among control, Cl_2 _treated or DMTU treated animals (Figure 7B). Lungs were removed 24 hours following Cl_2 _treatment for analysis of 4-HNE by GC-MS. Cl_2 _induced a significant increase in 4-HNE levels (Figure 7C). DMTU given either pre- or post- Cl_2 _treatment prevented any significant changes in 4-HNE levels (Figure 7C).

### Effects of Cl_2 _and DMTU treatments on GSH and GSSG intracellularly and extracellularly in the bronchoalveolar compartment

**Figure 8:**
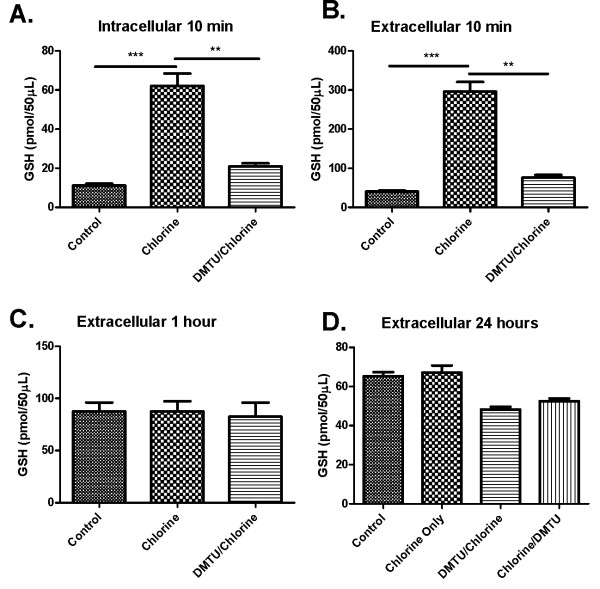
**Effects of Cl_2 _exposure and DMTU treatment on glutathione levels in BAL fluid and cells**. (A) 10 minutes following Cl_2 _exposure, GSH levels in the BAL cell fraction show a significant increase that was attenuated by pre-treating the mice with DMTU one hour prior to Cl_2 _challenge. (B) 10 minutes following Cl_2 _challenge, the same significant increase of GSH is seen in the BAL supernatant. (C) GSH levels 1 hour following Cl_2 _exposure and (D) 24 hours after Cl_2 _exposure were not different among groups. (n = 6-9; * p < 0.05, **p < 0.01, ***p < 0.001).

**Figure 9:**
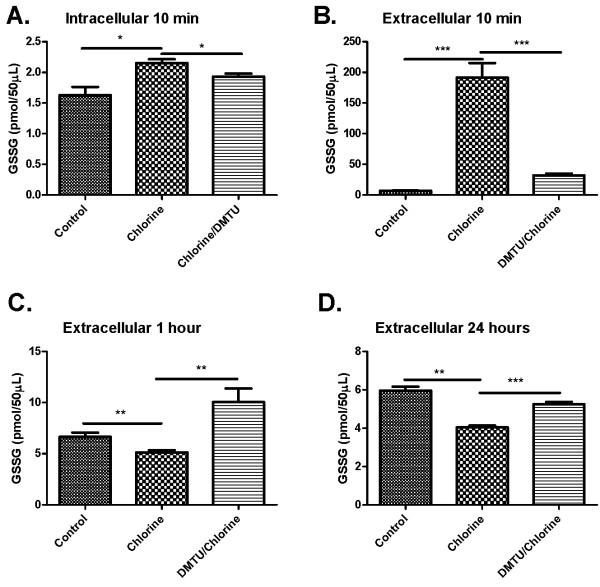
**Effects of Cl_2 _exposure and DMTU treatment on oxidized glutathione in BAL fluid cells and supernatant**. Ten minutes after Cl_2 _exposure (A-B), oxidized GSSG levels were determined. Animals exposed to Cl_2 _had increased GSSG in the BAL fluid and intracellularly 10 min following Cl_2 _exposure (A & B). Extracellular GSSG was reduced at one hour and 24 hours following Cl_2 _challenge, but no differences were found between control and DMTU treated groups.(n = 6-9; * p < 0.05, **p < 0.01, ***p < 0.001).

**Figure 10:**
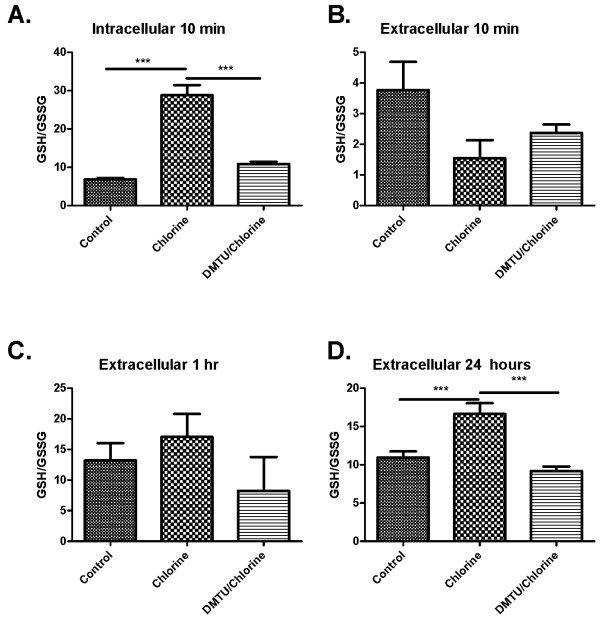
**Effect of Cl_2 _exposure and DMTU on ratio of GSH/GSSG**. (A) Ten minutes following Cl_2 _exposure the ratio of GSH/GSSG in the intracellular fraction of the BAL was significantly increased in Cl_2 _exposed mice compared to control and DMTU/Cl_2 _treated animals. (B-C) The extracellular fractions of the BAL at ten minutes and 1 hour showed no differences between groups. (D) Cl_2 _exposure induced a significant increase in the ratio of GSH/GSSG, an effect attenuated by DMTU. (n = 6-9; * p < 0.05, **p < 0.01, ***p < 0.001).

Cl_2 _increased both intracellular (Figure 8A) and extracellular (Figure 8B) GSH levels in BAL after 10 min, but had no effect on GSH levels after 1 and 24 hours (Figure 8C and 8D). Treatment with DMTU prior to administration of Cl_2 _blocked the increase in GSH in both compartments at 10 min (Figure 8A and 8B) but had no effect on GSH levels at the later time points (Figure 8C and 8D). Cl_2 _induced a significant increase in GSSG levels in the intracellular and extracellular compartments at 10 min (Figure 9A and 9B). At 1 and 24 hours there was a decrease in GSSG levels in Cl_2 _treated groups compared to control and DMTU treated groups that were restored by DMTU treatment (Figure 9C and 9D). The ratio of GSH/GSSG was significantly higher in the cell fraction of BAL in Cl_2 _exposed mice than control and DMTU treated mice at 10 minutes (Figure 10A). There was a trend towards a decrease in GSH/GSSG ratio in the extracellular compartment of the BAL at the same time point, but this was not statistically significant. Additionally, at 24 hours, the GSH/GSSG ratio remained high in the Cl_2 _treated mice but was attributable to a decline in GSSG at this time (Figure 10D). This effect was prevented by treatment with DMTU (Figure 10A and 10D).

## Discussion

In the current study we have shown that Balb/C mice exposed to Cl_2 _gas for 5 min develop concentration-dependent airway hyperresponsiveness to inhaled aerosolized MCh. At concentrations of Cl_2 _greater than 100 ppm there is evidence of epithelial damage with flattening of the cells and the shedding of ciliated cells into the bronchoalveolar lavage fluid. However, at a concentration of Cl_2 _(100 ppm), despite the lack of gross morphological changes in epithelial cells there was still a substantial degree of airway hyperresponsiveness, an effect potentially attributable to increased oxidative stress. The effect of Cl_2 _on airway function was attenuated by pre-treating the mice one hour before Cl_2 _exposure with an intraperitoneal injection of DMTU. Treatment with DMTU 1 hour after exposure to Cl_2 _also ameliorated the adverse effects on airway function. Oxidative injury to lung tissue was detected 24 hours post-Cl_2 _exposure and indicated by and increase lipid peroxidation in Cl_2 _exposed mice, an effect attenuated by pre- or post-Cl_2 _treatment with DMTU. Additionally, DMTU treatment maintained GSH/GSSG levels at those of control mice, whereas Cl_2 _only treated mice showed significant changes in both GSH and GSSG at various time points.

Airway hyperresponsiveness has been previously demonstrated to follow Cl_2 _exposure in both rat and mouse models of irritant induced asthma [15,16]. Pathological changes including airway remodeling occur following a single exposure to a high concentration of Cl_2 _in rats [17]. It seems likely that epithelial damage is a major contributor to the altered responsiveness to inhaled MCh. The epithelium could serve as a barrier that could reduce access of MCh to the smooth muscle or might attenuate the responsiveness to MCh through the release of relaxant substances such as NO or prostaglandins [18-20]. The mechanism of AHR following Cl_2 _may be similar to that of ozone in that both forms of injury are associated with oxidant damage to the tissues. Natural killer cells and interleukin-17 have been shown recently to be essential in the protection against airway damage and hyperresponsiveness following repeated ozone exposures [21]. Cl_2 _potentially causes toxicity through its highly reactive nature. However, it is also know to cause damage through the generation of hydrochloric acid (HCl). Indeed HCl has been shown to cause airway hyperresponsiveness in mice when administered into the airways, by mechanisms that have been suggested to relate to epithelial barrier function. However, it has been shown that HCl is much less toxic than Cl_2 _so it is likely that the effects of Cl_2 _induced oxidants are more likely to account for its adverse effects [22,7].

Irrespective of the mechanism of Cl_2 _induced airway hyperresponsiveness, DMTU was highly effective in preventing its development when given either as a pre-treatment or as a rescue treatment. Assuming that the therapeutic effects of DMTU are indeed mediated by anti-oxidant properties, the data suggest that the initial direct oxidative stress caused by Cl_2 _is only part of the oxidative burden and that another source of reactive oxygen is important in the time period between 1 and 24 h following Cl_2 _exposure. For example, secondary activation of neutrophils, macrophages or epithelium and various chemokines, cytokines and growth factors they secrete could conceivably contribute to airway damage in a mechanism similar those shown for respiratory viral infection [23].

Measures of oxidant injury such as nitric oxide production, as reflected in BAL nitrates/nitrites, and protein carbonylation were not detectably different from control animals at 24 hours after Cl_2 _exposure, consistent with a relatively mild injury compared to previous results [7]. However, presence of oxidative stress was apparent following assessment of lung tissue levels of 4-HNE, an indication of lipid peroxidation. 4-HNE levels were reduced to baseline by pre- and post-Cl_2 _treatment with DMTU, suggesting that lipid peroxidation is a prolonged effect of exposure to Cl_2 _further supporting the conclusion that the amelioration of markers of airway injury is likely mediated by anti-oxidant properties of DMTU.

Glutathione is an important endogenous antioxidant and changes in its intracellular and extracellular concentrations are expected following an oxidant challenge such as Cl_2_. Generally oxidant stress is noted to diminish GSH both intracellularly and extracellularly in the lung (reviewed in [24]) although glutathione increases as an adaptive response to oxidative stress associated for example with cigarette smoking or pulmonary infection [25,26]. We found that Cl_2 _exposure induced rapid and transient changes in glutathione concentrations. Ten minutes following exposure there was a surge in both intra- and extra-cellular GSH levels in BAL, presumably attributable to GSH synthesis and export into the extracellular milieu. Additionally, Cl_2 _may induce lysis of pulmonary cells, especially epithelial cells which might also contribute to the large amount of extracellular GSH. Epithelial cells are known to contain high levels of GSH [25] and high doses of Cl_2 _have been shown to cause epithelial cell shedding and/or lysis. However the changes in GSH observed in the current experiment occurred in the absence of significant changes in epithelial cell counts in BAL fluid or in epithelial cell numbers enumerated in the airway walls themselves. The changes in GSH were transient and had resolved by 1 hour. The rapid rise in GSH was prevented by pre-treatment with DMTU prior to Cl_2 _exposure, suggesting a measure of relief against the effects of oxidative stress.

In addition to the early spike in GSH concentration in BAL cells and fluid, we also noted a significant increase in GSH in its oxidized form, glutathione disulfide (GSSG), both intra-and exrtacellularly at 10 minutes, presumably indicative of oxidative stress in the lung. These changes were abrogated by DMTU supporting the idea that the mechanism of protection was through neutralization of oxygen metabolites. Furthermore, the protection provided by delayed treatment with DMTU further suggests that delayed oxidative stress is also a significant contributor to the response to injury. By 1 and 24 hours, GSH levels were restored but GSSG levels showed a significant decrease in chlorine exposed groups. It is not clear what the significance of this finding is for airway function. Despite the GSSG levels being depleted at this time point, the ratio of GSH/GSSG was higher in chlorine exposed mice compared with controls and DMTU treated animals. The anti-oxidants ascorbic acid, desferroxamine and N-acetyl-L-cysteine have been show to ameliorate the injury caused by Cl_2 _in the rat [9]. In these experiments there was evidence of depletion of GSH by Cl_2_, an observation that we have not confirmed. However the exposure in the rat was substantially greater (400 ppm for 30 minutes).

Consideration of oxidative stress as a target in irritant-induced asthma caused by potent oxidants is reasonable. However, oxidative stress-induced damage may also contribute to other forms of asthma. Asthmatic subjects manifest evidence of oxidative stress, as evidenced by a variety of changes including increased superoxide generation from leukocytes, increased total nitrites and nitrates, increased protein carbonyls, increased nitric oxide in exhaled breath condensate, increased lipid peroxidation products and decreased protein sulfhydryls in plasma [26]. They also show increased superoxide dismutase activity in red blood cells, increased total blood glutathione, and decreased glutathione peroxidase activity in red blood cells and leukocytes. A recent epidemiological study of childhood asthma demonstrated significant decreases in glutathione and amino acid precursors of glutathione as well as various other components of both enzymatic and non-enzymatic endogenous antioxidant defense mechanisms [27]. Thioredoxin, a reducing protein, may also inhibit experimental allergic asthma and airway remodeling [28].

In conclusion, exposure to modest levels of Cl_2 _(100 ppm) leads to an increase in airway responsiveness in mice. Mice exposed to Cl_2 _showed increases in total inflammatory cells, in particular neutrophils and lymphocytes. Despite lack of increases in nitrate/nitrite or carbonylated proteins, lipid peroxidation levels (4-HNE) were significantly higher in Cl_2 _exposed animals. Importantly, there was also evidence of a salutary treatment effect when DMTU was administered as late as 1 hour after the exposure to Cl_2 _suggesting that oxidative damage is an ongoing process following the initial injury. Treatment with anti-oxidants shortly after acute exposure to highly irritant oxidant substances such as Cl_2 _may have therapeutic utility.

## Competing interests

The authors declare that they have no competing interests.

## Authors' contributions

TM participated in the study design and performed the experiments for Figures 1, 2, 3, 4, 5, 6 in their entirety and harvested materials for analyses in Figures 7, 8, 9, 10. She also wrote the manuscript. WP contributed through the analyses of GSH and GSSG and assisted with the writing of the manuscript. BD and CW contributed to the revision of the paper and provided the analysis of 4-HNE. KG performed the measurements of NOx and approved the manuscript. HKQ provided critical review of the paper and assistance with data analysis. NL assisted in the setup of chlorine exposure and in supervising the exposure of animals in a safe manner. JJT assisted with analysis of biological samples. JGM was involved in the study design, in review of the data and in the preparation of the manuscript. All authors read and approved the final manuscript.
